# Assessing stress associated with temporomandibular joint disorder through Fonseca's anamnestic index among the Saudi physicians

**DOI:** 10.1002/cre2.157

**Published:** 2018-12-26

**Authors:** Samar O. Al Hayek, Mashael F. Al‐Thunayan, Amjad M. AlGhaihab, Reem M. AlReshaid, Aamir Omair

**Affiliations:** ^1^ College of Dentistry King Saud bin Abdulaziz University for Health Sciences, National Guard Hospital Riyadh Saudi Arabia; ^2^ College of Dentistry King Saud bin Abdulaziz University for Health Sciences Riyadh Saudi Arabia; ^3^ Dental College Riyadh Elm University Riyadh Saudi Arabia; ^4^ College of Medicine King Saud bin Abdulaziz University for Health Sciences Riyadh Saudi Arabia

**Keywords:** physicians, prevalence, Saudi Arabia, signs, temporomandibular joint disorders

## Abstract

The study aims to investigate signs and symptoms of temporomandibular disorders (TMD) among physicians in a tertiary health‐care center. It has estimated the level of symptomatology, determined the association with demographic data, and identified the related occupational risk factors. A cross‐sectional survey was used, and physicians of genders, all age groups, and nationalities from King Abdulaziz Medical City in Riyadh, Saudi Arabia, were recruited. Subjects who had rheumatic arthritis, osteoarthritis, trigeminal neuralgia, or temporomandibular joint (TMJ) trauma were excluded. The data were collected through a self‐administered questionnaire that measured TMD severity and oral parafunctional behaviors. Fonseca's anamnestic index (FAI) and an oral validated behavior checklist were used to assess the signs and symptoms of TMD. A total of 282 physicians participated in the study, and the prevalence of TMD signs among physicians was 37% (106); among them, 88 (83%) were within the light dysfunction category. Female physicians reported significantly higher FAI than males for side‐to‐side mandibular movement (12% vs. 5%, *P* = 0.04), reporting ear pain (18% vs. 10%, *P* = 0.04), and noticing clicking when chewing or opening the mouth (35% vs. 20%, *P* = 0.006). Younger practitioners (28–31 years old) who reported clicking while chewing or opening the mouth tended to have reported higher TMD dysfunction (35%) than those aged 40 and above (13%; *P* = 0.007). Self‐reported signs of TMD were 37% among our population. Information collected from FAI is useful in early diagnosis and prevention of TMD.

## 1 INTRODUCTION

Temporomandibular disorders (TMD) is a group of conditions that cause dysfunction in the temporomandibular joint leading to chronic recurrent pain along with its muscles and supporting tissues (McNeill, 1997). TMD has an effect not only on its sufferers but also on the community that endures its expensive treatment and poor productivity (Gatchel, Stowell, Wildenstein, Riggs, & Ellis, 2006). The signs indicating the probable presence or occurrence of a disease include limited jaw motions in vertical, lateral, and retrusive mandibular movements; deviation of the mandible; pain during some or all joint excursion; muscle pain (masseter, medial, and lateral pterygoids) during palpation; and joint clicking/crepitation sounds reported and palpated (Cooper & Kleinberg, 2007; Gøtzsche, 2007).

Symptoms of the disorder are clinical manifestations of temporomandibular joint pain that are sensed by the patient in a subjective manner, which is difficult to quantify (Gøtzsche, 2007). In a prevalence study of TMD, myofacial pain was found to be the most common diagnostic with a prevalence of 15% (Al‐Khotani et al., 2016). Other symptoms included jaw muscle stiffness, locking of the jaw, difficult or inadequate movement, chewing struggle, painful clicking of the joint during opening or closing the mouth, and change in teeth articulation (Cooper & Kleinberg, 2007). There are symptoms of TMD that do not encompass the musculoskeletal system such as nonotologic otalgia (ear pian that is not caused by the ear), dizziness, tinnitus, and toothache. TMD can also be manifested as tension headache, migraine, neck pain, and myofascial pain in that region, which may occur in combination or alone (Magnusson, Egermark, & Carlsson, 2005).

Every individual is subjective to certain factors of external environment and respond differently to the external stressors. It is important to identify the stresses and deal with it, appropriately. An important role is played by the psychological factors in the onset and development of TMD. Majority of the individuals activate their stomatognathic system to relieve their stresses by limiting teeth grinding, clenching teeth, and masticatory muscle contraction (Calixtre, Gruninger, Chaves, & Oliveira, 2014). The individuals suffering from TMD suffer from headaches that influence their functional and psychosocial quality of life. Previous literature investigated the presence of TMD among children, adolescent, and college students. TMD among physicians was not discussed thoroughly in literature before; nevertheless, depressive symptoms and stress among medical specialties have been recognized as risk factors for this disorder (Bernburg, Vitzthum, Groneberg, & Mache, 2016).

Majority of the previous studies have reviewed and addressed adolescents and children. Therefore, in the present epidemiologic study, self‐reported signs of temporomandibular joint disorders among physicians of a tertiary health‐care center have been evaluated. The aim of this study was to assess the prevalence of TMD signs among physicians and its association with oral parafunctional habits using Fonseca's anamnestic index (FAI). This association might highlight the eventual causative factors that would help in investigating the elimination of suspected factors and their relation with decreased signs of TMD. The main strength of the study was the inclusion of medical professionals from different areas of the hospital to compare between specialties, gender, and age group with respect to TMD signs and symptoms.

## 2 THEORETICAL BACKGROUND

TMD being a multifactorial etiology is associated with several factors that play an important role in the induction, perpetuation, and aggravation of TMD. Some of the previous studies have depicted that some symptoms and the pain intensity of TMD were higher among females (Bagis, Ayaz, Turgut, Durkan, & Özcan, 2012; Johansson, Unell, Carlsson, Söderfeldt, & Halling, 2003; Macfarlane, Blinkhorn, Davies, Kincey, & Worthington, 2002). Johansson et al. (2003) conducted a cross‐sectional study on 50‐year‐old subjects and yielded in TMJ pain prevalence of 7% and 12% in males and females, respectively. Another study done by distributing a self‐assessment survey on patients in a medical care center in England stated that orofacial pain prevalence of TMD was 21% in males and 30% in females (Macfarlane et al., 2002). The same study showed that pain was higher among the younger population (Macfarlane et al., 2002). In Jordan, more than two third of university students complained of at least one symptom of TMD (Ryalat et al., 2009). In a recent cross‐sectional study from Saudi Arabia conducted on children and adolescents, about one third of the participants were diagnosed with at least one TMD sign or symptom (Al‐Khotani et al., 2016). Another study assessed the prevalence of signs and symptoms of TMD and oral parafunction habits among Saudi adolescents (Feteih, 2006). The results showed that about one fifth and one third of the participants displayed at least one sign and symptoms of TMD, respectively. Moreover, females were found to be more affected as compared with males (Feteih, 2006).

Sign and symptoms of TMD can be assessed in different methods depending on the feasibility, time, and cost. Few of the studies have assessed the signs and symptoms of TMD by FAI because of its easy applicability comparing with other assessment tools. In Saudi Arabia, Habib et al. utilized this questionnaire to assess the reported signs and symptoms of TMD among the male university students. The previously mentioned research revealed that almost half of the participants reported of signs and symptoms of TMD (Fonseca, Bonfante, Valle, & Freitas, 1994; Habib et al., 2015).

Another study indicated an association between improper sleep, stress, and parafunctional habits in undergraduate and postgraduate dental students at Dow University of Health Sciences in Pakistan. The study showed that 56% of the students had stress‐related teeth grinding at night and stress was associated with improper sleep leading to parafunctional habits (Sardar, 2015). Van der Meulen, Lobbezoo, Aartman, and Naeije (2014) stated that there was no significant association between oral parafunctional habits and facial pain. However, oral parafunctional habits were assessed using oral behavior checklist (OBC) for its evident validity and reliability (Ohrbach, Beneduce, Markiewicz, & McCall, 2004; Van der Meulen et al., 2014).

## 3 MATERIAL AND METHODS

### 3.1 Study design

The study has employed an analytical cross‐sectional design. It was performed in different departments in National Guard Health Affairs (NGHA) medical center that belongs to the governmental sector in Riyadh, Saudi Arabia.

### 3.2 Study population

Saudi/non‐Saudi medical physicians of both genders of all age groups from the NGHA in Riyadh, Saudi Arabia, were selected for the study. The total study population was estimated to be 2,200 physicians, with a ratio of 3:1 male to females. The prevalence of TMD as found in the literature ranged between 7% and 30%. Assuming a prevalence of 30% (Al‐Khotani et al., 2016; Johansson et al., 2003), a population size of 2,200, a confidence level of 95%, and a precision of 5%, the optimal sample size was estimated to be 282 subjects. Sample size was calculated using the prevalence formula in nQuery software (Hodges & Pihlstrom, 1998). Subjects who had rheumatic arthritis, trigeminal neuralgia, or trauma to the TMJ were excluded.

### 3.3 Ethical considerations

Permission from the Executive Director of the Medical Services in NGHA was obtained to distribute the questionnaires. Moreover, IRB approval from King Abdullah International Medical Research Center was obtained before starting the data collection. In addition, an informed consent was signed from the participants for their participation in the study.

### 3.4 Data collection methods, instruments used, and measurements

Convenient sampling was used to approach towards all the available physicians in the wards or departments. The physicians were then given the questionnaire to be filled. The adopted self‐administered questionnaire is composed of three sections: (A) demographic data, (B) TMD severity, and (C) oral parafunctional behavior. Section A reported the social demographic data, participants' specialties, and years of practice since graduation.

Section B measured the TMD severity using FAI (Nomura et al., 2007). FAI composes of 10 questions with three options (yes, sometimes, and no) for each question. Each answer has its score with 2 = *yes*, 1 = *sometimes*, and 0 = *no*. One question was omitted in this questionnaire because of cultural difference because this research was conducted in Saudi Arabia. Total scores ranged from 0 to 18, which were categorized to no TMD (0–3), mild (4–8), moderate (9–13), or severe TMD (14–18; Fonseca et al., 1994; Nomura et al., 2007).

Section C was adopted from the Research Diagnostic Criteria for TMD questionnaire axis II. A validated OBC is used to better determine the presence of oral parafunctional behaviors, which are any abnormal behavior or functioning of the oral structures and associated muscles. OBC originally composes of 21 items; however, two items were omitted due to cultural reasons (Schiffman et al., 2014). The sum of scoring had the following scheme: none = 0, low = 1–16, and high = 17–76.

### 3.5 Statistical analysis

Data were analyzed using the Statistical Package for Social Sciences (SPSS 23 software, Chicago, IL, USA). Descriptive statistics were presented as categorical variables that are described in terms of frequencies and percentages (e.g., gender and specialty) and numerical variables that were described in terms of mean ± standard deviation (e.g., age and years of experience). With respect to age, the participants were divided into four groups based on the percentile‐derived intervals (25th percentile was 27 years, 50th percentile was 31 years, and 75th percentile was 39 years). Chi‐square test was used to compare categorical variables (e.g., OBC according to the presence of TMD dysfunction, gender and age group, and also TMD dysfunction with respect to gender, age group, and to specialties). A *P* value <0.05 was considered to show a significant association.

## 4 RESULTS

A total of 400 questionnaires were distributed; 282 (70.5%) were answered. The missing 118 included 51 questionnaires not returned, and 67 were excluded based on the exclusion criteria. There were 179 (64%) male and 103 (36%) female respondents. The mean age of the respondents was 33.9 ± 8.7 years, the majority (70%) of them having 1 to 10 years (median = 6 years) of practice since graduation (Table 1).

**Table 1:** Characteristics of the respondents (*N* = 282)

	Description	*n* (%)
Age (*n* = 281)	24 to 27 years	77 (27%)
	28 to 31 years	71 (25%)
	32 to 39 years	63 (22%)
	40+ years	70 (25%)
Years of practice since graduation (*n* = 279)	1 to 10 years	197 (70%)
	11 to 20 years	52 (18%)
	21 to 30 years	26 (9%)
	31 to 40 years	4 (1%)
Gender (*n* = 282)	Male	179 (64%)
	Female	103 (37%)

**Figure 1:**
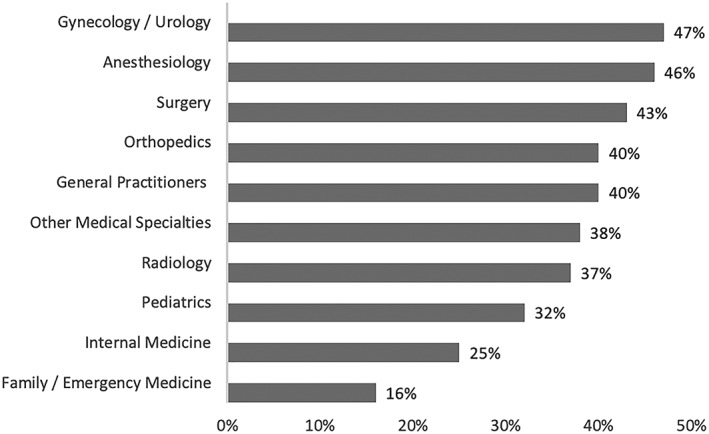
Prevalence of temporomandibular joint disorder among physicians by specialty (*N* = 282). Pearson chi‐square for temporomandibular disorders between different specialties: *P* value = 0.58

TMD dysfunction in this study was identified on the basis of self‐reported scores using the FAI (Fonseca et al., 1994; Nomura et al., 2007). According to FAI score, 176 (62%) of the participants did not have any signs of TMD dysfunction, whereas 88 (31%) had signs of light TMD dysfunction, 17 (6%) with moderate TMD dysfunction, and 1 (0.4%) with severe TMD dysfunction. TMD highest signs of dysfunction were among gynecology/urology (47%), followed by anesthesiology (46%), and surgery (43%), whereas the lowest was among internal medicine (25%) and family/emergency medicine (16%; Figure 1). Out of 146 of the respondents with high OBC, 76 (52%) of them had reported TMD dysfunction as compared with those with low OBC (*n* = 114) where only 27 (24%) reported TMD dysfunction and only three out of 22 of those who did report any parafunctional habits (14%) reported TMD dysfunction (Table 2).

**Table 2:** Comparison of severity of temporomandibular disorders based on classification of oral behavior checklist (*N* = 282)

		TMD severity classification based on FAI		
OBC classification	Total (*N* = 282)	With moderate to severe dysfunction (9–18) *n* = 18	Without dysfunction/light dysfunction (0–8) *n* = 264	*P* value
No	22 (8%)	0 (0%)	22 (100%)	0.005
Low (1–16)	114 (40%)	2 (2%)	112 (98%)	
High (17–76)	146 (52%)	16 (11%)	130 (89%)	

*Note*. TMD: temporomandibular disorders; FAI: Fonseca's anamnestic index; OBC: oral behavior checklist.

The comparison between respondents with signs of TMD dysfunction (*n* = 106) and those without TMD (*n* = 176) with the items on the OBC has been shown in Table 3. Most items on the OBC were found to be significantly associated with TMD dysfunction except for pressing tongue forcibly between teeth, using chewing gum, eating between meals, sustained talking, and holding the telephone between the head and shoulder.

**Table 3:** Association between oral behavior checklist and TMD dysfunction

Oral behavior checklist	Total (*n* = 282)	With TMD dysfunction (*n* = 106)	Without TMD dysfunction (*n* = 176)	*P* value
Clench or grind teeth when asleep, based on any information you may have	52	29 (27%)	23 (13%)	7
Sleep in a position that puts pressure on the jaw	117	61 (58%)	56 (32%)	<0.001
Grind teeth together during waking hours	37	25 (24%)	12 (7%)	<0.001
Clench teeth together during waking hours	59	38 (36%)	21 (12%)	<0.001
Press, touch, or hold teeth together other than while eating	76	45 (42%)	31 (18%)	<0.001
Hold, tighten, or tense muscles without clenching or bringing teeth together	40	21 (20%)	19 (11%)	0.03
Hold or jut jaw forward or to the side	31	18 (17%)	13 (7%)	0.01
Press tongue forcibly against teeth	29	15 (14%)	14 (8%)	0.09
Place tongue between teeth	33	19 (18%)	14 (8%)	0.01
Bite, chew, or play with your tongue, cheeks, or lips	78	40 (38%)	38 (22%)	0.003
Hold jaw in rigid or tense position	32	22 (21%)	10 (6%)	<0.001
Hold between the teeth or bite objects	52	31 (29%)	21 (12%)	<0.001
Use chewing gum	131	54 (51%)	77 (44%)	0.24
Lean with your hand on the jaw, such as cupping or resting the chin in the hand	103	49 (46%)	54 (31%)	0.009
Chew food on one side only	110	52 (49%)	58 (33%)	0.007
Eating between meals	146	58 (55%)	88 (50%)	0.44
Sustained talking	122	53 (50%)	69 (39%)	0.08
Yawning	139	62 (58%)	76 (43%)	0.01
Hold telephone between your head and shoulders	118	52 (49%)	66 (38%)	0.06

*Note*. Those who answered “yes” and “sometimes” were grouped into one category (with TMD dysfunction). Those who answered “>1 night/month” were considered as yes. TMD: temporomandibular disorders.

The respondents were categorized into four age groups according to the quartiles (24–27, 28–31, 32–39, and 40+ years). Table 4 has shown the items on the OBC that were found to be significantly associated with age group. It was found that respondents in the older age group of 40+ years had relative lower percentages of reporting on the OBC as compared with those in the younger age groups.

**Table 4:** Oral behavior checklist factors of respondents who reported presence of the habit at least one night a month showing significant difference by age quartiles

	Age quartiles				
Oral behavior checklist	Q1 (24–27 years; *n* = 77)	Q2 (28–31 years; *n* = 71)	Q3 (32–39 years; *n* = 63)	Q4 (40+ years; *n* = 70)[Link]	*P* value
Sleep in a position that puts pressure on the jaw	36 (47%)	34 (48%)	29 (46%)	17 (24%)	0.01
Clench teeth together during waking hours	18 (23%)	21 (30%)	14 (22%)	6 (9%)	0.01
Press, touch, or hold teeth together other than while eating	27 (35%)	23 (32%)	17 (27%)	9 (13%)	0.01
Hold, tighten, or tense muscles without clenching or bringing teeth together	12 (16%)	17 (24%)	8 (13%)	3 (4%)	0.009
Bite, chew, or play with your tongue, cheeks, or lips	25 (33%)	28 (39%)	15 (24%)	10 (14%)	0.006
Hold between the teeth or bite objects	18 (23%)	13 (18%)	16 (25%)	5 (7%)	0.02
Use chewing gum	44 (57%)	37 (52%)	33 (52%)	17 (24%)	<0.001
Lean with your hand on the jaw	38 (49%)	29 (41%)	24 (38%)	12 (17%)	<0.001
Eating between meals	45 (58%)	41 (58%)	34 (54%)	26 (37%)	0.03
Yawning	46 (60%)	36 (51%)	33 (52%)	23 (33%)	0.01
Hold telephone between your head and shoulders	35 (46%)	34 (48%)	31 (49%)	17 (24%)	0.008

Quartile 4 percentages were significantly less than the other three quartiles.

The most commonly reported symptom in this study was stress (53%) followed by headache (42%) and neck/nape pain (31%). Female physicians (*n* = 103) were more likely to report symptoms on the FAI as compared with males (*n* = 179) regarding moving the mandible side to side (12% vs. 5%, *P* = 0.04), feeling pain in the ear of the temporomandibular joint (18% vs. 10%, *P* = 0.04), and noticing clicking when chewing or opening the mouth (35% vs. 20%, *P* = 0.006). Younger practitioners (28–31 years old) who reported clicking while chewing or opening the mouth tend to have reported TMD dysfunction based on FAI scores more than those aged 40 years and above (35% vs. 13%, *P* = 0.007).

**Figure 2:**
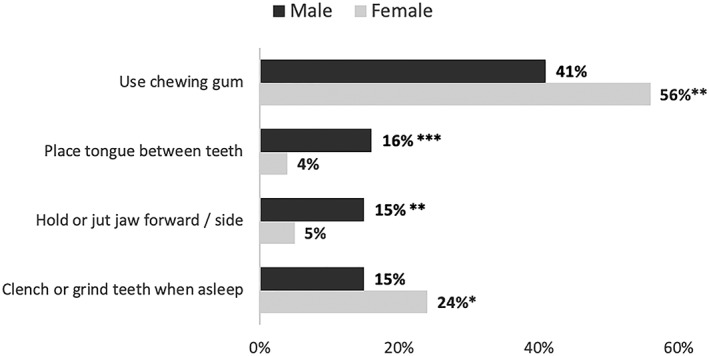
Frequency and percentage of oral behavior checklist according to gender (males = 179, females = 103). Pearson chi‐square significance for oral behavior checklist according to gender: ^*^ *P* = 0.06, ^**^ *P* = 0.01, ^***^ *P* < 0.01

Figure 2 has illustrated the significant differences in OBC items based on gender differences. A greater proportion of females, that is, 58 (56%) out of 103, reported the use of chewing gum as compared with 73 (41%) out of 179 males (*P* = 0.01). The males (16%) were more likely to report placing the tongue between teeth as compared with 4% of the females (*P* = 0.002) and jutting the jaw forward (15% vs. 5%, *P* = 0.01).

## 5 DISCUSSION

The present study was conducted in one of the main tertiary health‐care hospitals in Riyadh to assess the self‐reported signs of TMD among physicians and its associated signs and symptoms using FAI. King Abdul‐Aziz Medical City is known to have highly qualified physicians and high number of patients, and providing good quality care to a high number of patients is a difficult and stressful responsibility. The association of TMD signs and symptoms to stress has been discussed and linked with some of the previous studies (Habib et al., 2015; LeResche, Mancl, Drangsholt, Saunders, & Von Korff, 2005; Sardar, 2015; Yap et al., 2003).

In the present study, females had significantly higher FAI values for moving the mandible side to side, having tinnitus or pain related to the temporomandibular joint, and noticing clicking when chewing or opening the mouth. Similar results were reported in a study in Brazil associating between age and gender with TMD symptoms concluded that higher chances of presenting pain symptoms and dysphonia were more in the female group than the male (Ferreira, Silva, & Felício, 2016). The orofacial pain distribution regarding gender especially in the TMD suggested a possible association between the female sex hormones or the pain modulation mechanisms and the TMD, acknowledging that women are more sensitive to pain modalities (LeResche, Mancl, Sherman, Gandara, & Dworkin, 2003; McEwen, Alves, Bulloch, & Weiland, 1998; Sarlani, Garrett, Grace, & Greenspan, 2007). A study similar to the present analysis reported prevalence of male students in Riyadh who reported TMD signs and symptoms using FAI. The results showed that 10% of the respondents reported moderate to severe TMD (Habib et al., 2015). In the present study, almost one third of the physicians were found to have one or more of TMD symptoms, with 6% of them in the moderate to severe category. The prevalence could have been higher if the study included physicians with osteoarthritis, rheumatic arthritis, trigeminal neuralgia, or any trauma to the temporomandibular joint.

The present study has identified three specialties to report the highest TMD signs and symptoms including gynecology/urology, anesthesiology, and surgery. Previous studies have reported the risk factors of the TMD such as stress and depressive symptoms. For instance, a study conducted in German hospitals showed that psychosocial distress was highest in surgical medicine whereas depressive symptoms were highest in internal medicine followed by gynecology specialty (Bernburg et al., 2016). The findings regarding the patterns for TMD age‐related signs and symptoms reported were consistent with results of the present study (Manfredini, Piccotti, Ferronato, & Guarda‐Nardini, 2010). Reporting any clicking while opening or closing the mouth was common among population younger than 40 years old. Manfredini et al. (2010) reported an interesting finding in which the mean age of TMD with a diagnosis of disc displacement with or without pain was 32.7 years, whereas inflammatory disorders (osteoarthritis/osteoarthrosis) had a mean age of 54.2 years. This made clear that the patients with symptoms of a clicking sound accompanied with pain were mostly young aged (Manfredini et al., 2010).

A similar study conducted among university students in Riyadh showed that one third of males reported the presence of psychological stress (Habib et al., 2015). However, the present study has revealed that more than half of the respondents were stressed. The high percentage of stress that is reported by both genders in the present study is evidence of the heavy workload that is experienced by the physicians in NGHA. On the contrary, a study done in the United Kingdom concluded that both age and sex of medical doctors were not significant when measuring stress, which is associated with TMD (Goodfellow, Varnam, Rees, & Shelly, 1997; Sardar, 2015). Nonetheless, the same study concluded that the job itself for a physician is not the cause of stress. Oral parafunctional habits would contribute to TMD onset and could be considered a risk factor if only a score in the 17–76 range is reported from the OBC (Schiffman et al., 2014). Occurrence of these habits increases the probability of having one or more TMD sign and symptoms. Half of the physicians who reported TMD dysfunction were categorized in the high oral parafunctional habits; likewise, a study in São Paulo, Brazil, on adolescents identified sleep bruxism, awake bruxism, and other parafunctional habits to have TMD pain (Fernandes, van Selms, Gonçalves, Lobbezoo, & Camparis, 2015).

The results of present study revealed that the habit of holding objects between teeth or biting objects, grinding, or clenching the teeth has been significantly associated with signs of temporomandibular joint dysfunction. Winocur, Littner, Adams, and Gavish (2006) found similar results on association of oral habits and signs and symptoms of TMD for adolescents. Their results revealed significant association of bruxism to several TMD signs and symptoms including joint disturbances, pain/fatigue during chewing, and joint sensitivity to palpation (Winocur et al., 2006). The present study has depicted that leaning with the hand on the jaw was significantly associated with reported TMD whereas continuous arm leaning has significant association with both pain/fatigue during chewing and joint sensitivity to palpation (Winocur et al., 2006). Another study has found a significant association between harmful oral habits such as lip, object biting, grinding, and clenching of the teeth with signs and symptoms of TMD (Motta et al., 2013).

The present study has assessed the prevalence of TMD signs and symptoms among physicians and its association with oral parafunctional habits. The study concluded that the reporting of TMD among physicians was found to be 37%. Moreover, about one third of these had mild TMD dysfunction according to FAI. The study also revealed that the OBC features were significantly greater in the younger age groups as compared with the 40+‐year age group. More than half of the physicians had high frequency of oral parafunctional habits, with the greatest proportion among gynecologists/urologists, surgeons, and anesthesiologists. A number of factors were identified on the OBC that was significantly higher among those who reported as having TMD dysfunction.

Some possible related factors such as malocclusion were not investigated in this study because they needed clinical examination. Moreover, the signs of TMD dysfunction were based on self‐reported questionnaire (FAI). Another study limitation is the exclusion of factors that were concerned to cultural aspects; therefore, future studies need to incorporate those questions to evaluate the impact of cultural factors on TMD. Further research is needed to investigate other TMD predisposing variables for the physicians and expand the study to include clinical examination to support the diagnosis of TMD.

## DISCLOSURE

This study was approved by institutional review board of King Abdullah International Medical Research Center (KAIMRC).

## Funding Information

No funding information provided.

## CONFLICT OF INTEREST

None declared.

## References

[cre2157-bib-0001] Al‐Khotani, A. , Naimi‐Akbar, A. , Albadawi, E. , Ernberg, M. , Hedenberg‐Magnusson, B. , & Christidis, N. (2016). Prevalence of diagnosed temporomandibular disorders among Saudi Arabian children and adolescents. The Journal of Headache and Pain, 17(1), 41 10.1186/s10194-016-0642-9 27102118 PMC4840132

[cre2157-bib-0002] Bagis, B. , Ayaz, E. A. , Turgut, S. , Durkan, R. , & Özcan, M. (2012). Gender difference in prevalence of signs and symptoms of temporomandibular joint disorders: A retrospective study on 243 consecutive patients. International Journal of Medical Sciences, 9(7), 539–544. 10.7150/ijms.4474 22991492 PMC3444974

[cre2157-bib-0003] Bernburg, M. , Vitzthum, K. , Groneberg, D. A. , & Mache, S. (2016). Physicians' occupational stress, depressive symptoms and work ability in relation to their working environment: A cross‐sectional study of differences among medical residents with various specialties working in German hospitals. BMJ Open, 6(6). e011369: 10.1136/bmjopen-2016-011369 PMC4916614 27311909

[cre2157-bib-0004] Calixtre, L. B. , Gruninger, B. L. D. S. , Chaves, T. C. , & Oliveira, A. B. D. (2014). Is there an association between anxiety/depression and temporomandibular disorders in college students? Journal of Applied Oral Science, 22(1), 15–21. 10.1590/1678-775720130054 24626244 PMC3908760

[cre2157-bib-0005] Cooper, B. C. , & Kleinberg, I. (2007). Examination of a large patient population for the presence of symptoms and signs of temporomandibular disorders. CRANIO®, 25(2), 114–126. 10.1179/crn.2007.018 17508632

[cre2157-bib-0006] Fernandes, G. , van Selms, M. K. , Gonçalves, D. A. D. G. , Lobbezoo, F. , & Camparis, C. M. (2015). Factors associated with temporomandibular disorders pain in adolescents. Journal of Oral Rehabilitation, 42(2), 113–119. 10.1111/joor.12238 25244610

[cre2157-bib-0007] Ferreira, C. L. P. , Silva, M. A. M. R. D. , & Felício, C. M. D. (2016). Signs and symptoms of temporomandibular disorders in women and men In Codas (Vol. 28, No. 1, pp. 17‐21). São Paulo: Sociedade Brasileira de Fonoaudiologia. 27074184 10.1590/2317-1782/20162014218

[cre2157-bib-0008] Feteih, R. M. (2006). Signs and symptoms of temporomandibular disorders and oral parafunctions in urban Saudi Arabian adolescents: A research report. Head & Face Medicine, 2(1), 25 10.1186/1746-160X-2-25 16914032 PMC1563458

[cre2157-bib-0009] Fonseca, D. M. D. , Bonfante, G. , Valle, A. L. D. , & Freitas, S. F. T. D. (1994). Diagnóstico pela anamnese da disfunção craniomandibular. RGO, 23–28.

[cre2157-bib-0010] Gatchel, R. J. , Stowell, A. W. , Wildenstein, L. , Riggs, R. , & Ellis, E. III (2006). Efficacy of an early intervention for patients with acute temporomandibular disorder–related pain: A one‐year outcome study. The Journal of the American Dental Association, 137(3), 339–347. 10.14219/jada.archive.2006.0183 16570467

[cre2157-bib-0011] Goodfellow, A. , Varnam, R. , Rees, D. , & Shelly, M. P. (1997). Staff stress on the intensive care unit: A comparison of doctors and nurses. Anaesthesia, 52(11), 1037–1041. 10.1111/j.1365-2044.1997.213-az0348.x 9404163

[cre2157-bib-0012] Gøtzsche, P. (2007). Rational diagnosis and treatment: Evidence‐based clinical decision‐making John Wiley & Sons.

[cre2157-bib-0013] Habib, S. R. , Al Rifaiy, M. Q. , Awan, K. H. , Alsaif, A. , Alshalan, A. , & Altokais, Y. (2015). Prevalence and severity of temporomandibular disorders among university students in Riyadh. Saudi Dent J., 27(3), 125–130. 10.1016/j.sdentj.2014.11.009 26236125 PMC4501441

[cre2157-bib-0014] Hodges, J. S. , & Pihlstrom, B. L. (1998). Software support for clinical studies: Review of nQuery Advisor release 2.0. Journal of Dental Research, 77(3), 525–526. 10.1177/00220345980770031201 9496926

[cre2157-bib-0015] Johansson, A. , Unell, L. , Carlsson, G. E. , Söderfeldt, B. , & Halling, A. (2003). Gender difference in symptoms related to temporomandibular disorders in a population of 50‐year‐old subjects. Journal of Orofacial Pain, 17(1), 231–237. 10.1080/00016350410001649 12756928

[cre2157-bib-0016] LeResche, L. , Mancl, L. , Sherman, J. J. , Gandara, B. , & Dworkin, S. F. (2003). Changes in temporomandibular pain and other symptoms across the menstrual cycle. Pain, 106(3), 253–261. 10.1016/j.pain.2003.06.001 14659508

[cre2157-bib-0017] LeResche, L. , Mancl, L. A. , Drangsholt, M. T. , Saunders, K. , & Von Korff, M. (2005). Relationship of pain and symptoms to pubertal development in adolescents. Pain, 118(1–2), 201–209. 10.1016/j.pain.2005.08.011 16213087

[cre2157-bib-0018] Macfarlane, T. V. , Blinkhorn, A. S. , Davies, R. M. , Kincey, J. , & Worthington, H. V. (2002). Oro‐facial pain in the community: Prevalence and associated impact. Community Dentistry and Oral Epidemiology, 30(1), 52–60. 10.1034/j.1600-0528.2002.300108.x 11918576

[cre2157-bib-0019] Magnusson, T. , Egermark, I. , & Carlsson, G. E. (2005). A prospective investigation over two decades on signs and symptoms of temporomandibular disorders and associated variables. A final summary. Acta Odontologica Scandinavica, 63(2), 99–109. 10.1080/00016350510019739 16134549

[cre2157-bib-0020] Manfredini, D. , Piccotti, F. , Ferronato, G. , & Guarda‐Nardini, L. (2010). Age peaks of different RDC/TMD diagnoses in a patient population. Journal of Dentistry, 38(5), 392–399. 10.1016/j.jdent.2010.01.006 20100537

[cre2157-bib-0021] McEwen, B. S. , Alves, S. E. , Bulloch, K. , & Weiland, N. G. (1998). Clinically relevant basic science studies of gender differences and sex hormone effects. Psychopharmacology Bulletin, 34(3), 251. 9803750

[cre2157-bib-0022] McNeill, C. (1997). History and evolution of TMD concepts. Oral Surgery, Oral Medicine, Oral Pathology, Oral Radiology and Endodontics, 83(1), 51–60. 10.1016/S1079-2104(97)90091-3 9007924

[cre2157-bib-0023] Motta, L. J. , Guedes, C. C. , De Santis, T. O. , Fernandes, K. P. , Mesquita‐Ferrari, R. A. , & Bussadori, S. K. (2013). Association between parafunctional habits and signs and symptoms of temporomandibular dysfunction among adolescents. Oral Health & Preventive Dentistry, 11(1), 3–7. 23507675 10.3290/j.ohpd.a29369

[cre2157-bib-0024] Nomura, K. , Vitti, M. , Oliveira, A. S. D. , Chaves, T. C. , Semprini, M. , Siéssere, S. , … Regalo, S. C. H. (2007). Use of the Fonseca's questionnaire to assess the prevalence and severity of temporomandibular disorders in Brazilian dental undergraduates. Brazilian Dental Journal, 18(2), 163–167. 10.1590/s0103-64402007000200015 17982559

[cre2157-bib-0025] Ohrbach, R. , Beneduce, C. , Markiewicz, M. , & McCall, W. Jr. (2004). Psychometric properties of the oral behaviors checklist: Preliminary findings. J Dent Res, 83(special issue A), 1194.

[cre2157-bib-0026] Ryalat, S. , Baqain, Z. H. , Amin, W. M. , Sawair, F. , Samara, O. , & Badran, D. H. (2009). Prevalence of temporomandibular joint disorders among students of the University of Jordan. Journal of Clinical Medicine Research, 1(3), 158. 22493650 10.4021/jocmr2009.06.1245 PMC3318879

[cre2157-bib-0027] Sardar, K. P. (2015). Association among improper sleep, stress and parafunctional habits in dental students at Dow University of Health Sciences. JPDA, 24(04), 194.

[cre2157-bib-0028] Sarlani, E. , Garrett, P. H. , Grace, E. G. , & Greenspan, J. D. (2007). Temporal summation of pain characterizes women but not men with temporomandibular disorders. Journal of Orofacial Pain, 21(4), 309–317. 18018992 PMC5086416

[cre2157-bib-0029] Schiffman, E. , Ohrbach, R. , Truelove, E. , Look, J. , Anderson, G. , Goulet, J. P. , … Svensson, P. (2014). Diagnostic criteria for temporomandibular disorders (DC/TMD) for clinical and research applications: Recommendations of the International RDC/TMD Consortium Network and Orofacial Pain Special Interest Group. Journal of oral & facial pain and headache, 28(1), 6 10.11607/jop.1151 24482784 PMC4478082

[cre2157-bib-0030] Van der Meulen, M. J. , Lobbezoo, F. , Aartman, I. H. A. , & Naeije, M. (2014). Validity of the oral behaviours checklist: Correlations between OBC scores and intensity of facial pain. Journal of Oral Rehabilitation, 41(2), 115–121. 10.1111/joor.12114 24274580

[cre2157-bib-0031] Winocur, E. , Littner, D. , Adams, I. , & Gavish, A. (2006). Oral habits and their association with signs and symptoms of temporomandibular disorders in adolescents: A gender comparison. Oral Surgery, Oral Medicine, Oral Pathology, Oral Radiology, and Endodontology, 102(4), 482–487. 10.1016/j.tripleo.2005.11.007 16997115

[cre2157-bib-0032] Yap, A. U. , Dworkin, S. F. , Chua, E. K. , List, T. , Tan, K. B. , Prosthodont, C. , & Tan, H. H. (2003). Prevalence of temporomandibular disorder subtypes, psychologic distress, and psychosocial dysfunction in Asian patients. Journal of Orofacial Pain, 17(1). 12756927

